# “Using the same hand”: The complex local perceptions of integrated one health based interventions in East Africa

**DOI:** 10.1371/journal.pntd.0010298

**Published:** 2022-04-04

**Authors:** Alicia Davis, Jennika Virhia, Catherine Bunga, Shayo Alkara, Sarah Cleaveland, Jonathan Yoder, Safari Kinung’hi, Felix Lankester

**Affiliations:** 1 Institute of Health and Wellbeing, School of Social and Political Sciences, University of Glasgow, Glasgow, United Kingdom; 2 National Institute for Medical Research (NIMR), Mwanza Centre, Mwanza, Tanzania; 3 Global Animal Health Tanzania, Arusha, Tanzania; 4 Institute of Biodiversity Animal Health and Comparative Medicine, University of Glasgow, Glasgow, United Kingdom; 5 Paul G. Allen School for Global Health, Washington State University, Pullman, Washington, United States of America; Instituto de Salud Carlos III, SPAIN

## Abstract

**Background:**

Neglected Tropical Diseases (NTDs) such as soil transmitted helminths (STH) and human rabies represent a significant burden to health in East Africa. Control and elimination remains extremely challenging, particularly in remote communities. Novel approaches, such as One Health based integrated interventions, are gaining prominence, yet there is more to be learned about the ways in which social determinants affect such programmes.

**Methodology:**

In 2015 a mixed method qualitative study was conducted in northern Tanzania to determine community perceptions towards integrated delivery of two distinct healthcare interventions: treatment of children for STH and dog vaccination for rabies. In order to assess the effectiveness of the integrated approach, villages were randomly allocated to one of three intervention arms: i) **Arm A** received integrated mass drug administration (MDA) for STH and mass dog rabies vaccination (MDRV); ii) **Arm B** received MDA only; iii) **Arm C** received MDRV only.

**Principle findings:**

Integrated interventions were looked upon favourably by communities with respondents in all arms stating that they were more likely to either get their dogs vaccinated if child deworming was delivered at the same time and vice versa. Participants appreciated integrated interventions, due to time and cost savings and increased access to essential health care. Analysis of qualitative data allowed deeper exploration of responses, revealing *why* people appreciated these benefits as well as constraints and barriers to participation in integrated programmes.

**Conclusions/significance:**

An interdisciplinary One Health approach that incorporates qualitative social science can provide key insights into complex local perceptions for integrated health service delivery for STH and human rabies. This includes providing insights into how interventions can be improved while acknowledging and addressing critical issues around awareness, participation and underlying health disparities in remote pastoralist communities.

## Author summary

One Health approaches focus on human, animal, and environmental health and can offer a viable framework for integrated health service delivery for neglected tropical and other endemic diseases. In this study, an integrated human and animal health intervention to control both soil transmitted helminths (STH) in humans and rabies in dogs (and thereby humans) was viewed favourably by communities and suggested integrated interventions could result in increased participation. However, qualitative social science evaluation of this approach demonstrates that there are issues that may affect participation in integrated health interventions, such as local perceptions regarding hygiene issues where human and animal health are integrated. Qualitative social science research reveals that integrated interventions are more complex than at first glance and that ‘cost reduction’ should not just be the central motivation of such interventions.

## Introduction

*“Combining the delivery of multiple health interventions has the potential to minimize costs and expand intervention coverage*.*”*

[Kabatereine, 2010: p1, 1]

*“Indeed, many of the concepts currently in vogue in public health—from ‘cost effectiveness’ to ‘sustainability’ and ‘replicability’–are likely to be perverted unless social justice remains central to public health and medicine”*

[Farmer, 2005: p.18-19, 2]

Two particular neglected tropical diseases (NTD) have profound effects on human health in East Africa, soil-transmitted helminths (STH) [3–6] and canine mediated human rabies (human rabies) [7–8]. While the term “tropical” is often rightly critiqued in social sciences for its colonial and political connotations [9] we continue to use the term NTD primarily because of its current integration and widespread use in global and national health programmes. In this study we explored novel means to deliver health services for these two NTDs by combining interventions for STH and rabies in northern Tanzania.

STH are a group of parasitic nematode worms (including roundworms (*Ascaris lumbricoides*), whipworms (*Trichuris trichuria*) and hookworms (*Necator americanus*)) that infect more than 1.5 billion (24%) of the world’s population [3]. The parasite eggs contaminate soil, food, and water and expose vulnerable people, particularly children, to infection through ingestion of eggs via dirty hands and contaminated or uncooked food or water, or via skin penetration of larvae. Treatment for those infected with STH can usually be provided through a single dose of an anthelmintic drug such as albendazole or mebendazole. However, if left untreated, STH can cause both short and long-term health impacts including malnutrition, pregnancy complications and low birth weight, and developmental delays (cognitive and physical) [10–12] [4]. In areas with other severe disease problems such as HIV/AIDS, TB, or malaria, STH can suppress immune systems, increasing susceptibility to these life-threatening maladies [6] [13–14]. Consequently, the benefits associated with STH control are considerable.

The use of mass drug administration (MDA), typically targeting school aged-children at six-month intervals [15], has been an effective tool at reducing the burden of STH infections particularly in rural communities across Africa [1]. However, constraints exist due to cost, poor school attendance, and a lack of reach of susceptible community members who do not attend school, such as adults and unenrolled children [16].

Human rabies is a neglected zoonotic disease caused by bites from rabid animals, most commonly dogs [17]. Human rabies causes approximately 59,000 deaths and 8.6 billion USD in economic losses annually with the highest incidence occurring in rural African communities [8] [7]. Effective vaccines are available and can either be used to control rabies at the source through the vaccination of domestic dogs or to protect people bitten by rabid dogs through post-exposure prophylaxis (PEP) [18]. Controlling rabies at the source requires high levels of dog vaccination coverage (70%) to be achieved consistently across the vast areas of rural Africa where rabies remains endemic [19]. Achieving this level of coverage however, can be difficult and costly. For example, low coverage, even in annual campaigns, frequently occurs due to low dog owner turnout, poor event communication and notification, conflicting time constraints and priorities, and opportunity costs (i.e. cost of attending an intervention rather than engaging in other activities) [20].

Despite calls to fully eliminate NTDs like STH and rabies by 2030 [21–22], controlling these infections, particularly in remote rural communities in Africa, remains extremely challenging. Global health organisations, national governments and NGOs have thus stepped up efforts to address health concerns from NTDs through the use of novel interventions [16]. Yet, studies in African settings reveal that the determinants for the effectiveness of care for NTDs are multiple and complex [23]. Community level treatments for child illness, for example, “hinge on the myriad decisions that families face” about illness as well as on other constraints to their choices and access to care (Colvin et al., 2013: p. 67 [23]) for example infrastructural barriers, household economics, or decision making power. There is therefore the need for the inclusion of social science in the delivery and evaluation of health programmes to highlight how uptake is influenced by broader contextual social processes [24]. In light of this, practitioners have called for integrating social and biological sciences in order to address broader socio-political and economic challenges and the complex contexts within which NTDs exist [24–25]. Thus, in tackling how best to control and reduce NTDs, researchers and health practitioners have attempted to create new platforms through the integration of existing and successful delivery programmes, sometimes in creative ways [16] [26–27].

### Social science, One health and NTDs

*“‘One Health’ approaches argue for an integrated*, *holistic approach*. *Understanding intersections between disease dynamics*, *environmental drivers*, *livelihood systems and veterinary and public health responses is essential*.*”*

*-* [Scoones, 2017: p. 1] [28]

One Health (OH), the concept of the inter-linkages between human, animal, and ecosystem health [29–31] has gained prominence as a theoretical and practical approach to tackling complex disease challenges, and in particular zoonotic diseases, like rabies (see Rock et al. 2017 [32]), where the daily interaction between humans and animals can facilitate disease transmission. A One Health framework can support innovation and experimentation by combining interventions or treatments which can improve care providers’ and households’ options [23]. This is particularly important in difficult circumstances where people (and their animals) face multiple health risks and yet are systematically constrained in the decisions they can make. There are few instances where the One Health approach has been implemented through integrated animal–human health interventions, however examples of this approach include the prevention of zoonotic disease [33] and provision of vaccination services [34]. Thus, integrated and cost-effective health care delivery programmes that do not force people to choose between their own health or that of their livestock should provide an example of the benefits of One Health [16].

One Health not only calls for the integration of animal, human and environmental health but also offers space to integrate disciplines as a means for applying unique approaches to complex human and animal health problems [35]. The social sciences have an important role to play in understanding and implementing One Health approaches, particularly qualitative social science [36–40] (see special issues of SSM 2015 or Med Anthro Quart 2019 for rich discussion of these contributions). Qualitative research can improve intervention response by identifying people’s preferences, developing collaborative frameworks that co-produce valuable knowledge, and by promoting and supporting transparent public engagement and education [36] [38]. Qualitative social science can also bring in broader socio-political and cultural contexts which impact health [38–39]. In this matter, our own ethnographic research on zoonoses in Tanzania, has revealed otherwise ignored emergent diseases of concern, such as coenurosis in small ruminant livestock caused by the tapeworm *Taenia multiceps*; exposed the efforts people are taking to tackle disease risks; revealed the socio-political and economic constraints people face to improve health, and explores why people may or may not change health-impacting behaviours [35] [41–42].

Understanding broad contextual and social factors are important for the studies of NTDs as they not only impact health outcomes but can also create (in)equitable health landscapes, particularly where those left out of interventions are disadvantaged. People who are more likely to find themselves suffering from NTDs are also more likely to find themselves unable to access adequate therapy to treat these diseases [43]. Health inequities, often experienced most by the impoverished and disadvantaged within and across societies, can be considered ‘structural violence’ [44] [2]. Identifying key areas within health systems for equitable solutions, targeting disadvantaged populations is essential if more people (and animals and the environment) are to be reached and health outcomes improved, and not just for some. Applying a careful One Health approach, with particular attention to social processes, could help draw attention to structural and contextual challenges which may influence perceptions of, participation in, and uptake of intervention programmes [45]. We consider that qualitative social science (and One Health when applicable) be viewed as a central component in addressing NTDs in order to facilitate more effective disease control interventions that improve public and animal health.

This study utilised such an approach to bring people and animals together for the delivery and investigation of interventions to prevent two epidemiologically unrelated health problems, STH and rabies. It was the independent popularity of the two distinct health campaigns which suggested that the coupling of these two NTDs would be feasible and created the rare opportunity to explore integration of One Health based delivery platforms and understand public opinion, social contexts, and awareness about each. Lankester et al. 2019 [16] described the integrated intervention, the non-integrated delivery interventions that were used for comparison, and the coverage and cost comparisons made. In this paper we used a mixed methods approach, with a particular focus on qualitative data, to describe the community perceptions and opinions regarding integrated health delivery and the role that this delivery strategy might have in the control of NTDs. As the quotes at the start of this paper suggest, we aim to highlight the social benefits beyond cost-effectiveness of the linked human-animal approach.

## Methods

The study was carried out between January and September 2015 in the Ngorongoro District of northern Tanzania, a remote area situated on the eastern boundary of the Serengeti National Park that is predominantly inhabited by Maasai transhumant pastoralists (e.g. herders who migrate seasonally, but only with their livestock). Migrations in the past several decades have been increasingly localised and access to grazing areas increasingly restricted to ‘local’ residents (in part due to land loss, drought, and other land management issues), however boundaries for grazing do maintain some degree of flexibility (particularly during drought conditions) [35]. Maasai communities in Ngorongoro District look much like pastoral and agro-pastoral areas throughout northern and central Tanzania and southern Kenya with permanent villages and homesteads, schools, village offices, shops, farms (in some villages) and a relatively poorly resourced human and veterinary health service infrastructure [41]. The area was chosen because mass dog rabies vaccination (MDRV) and MDA are both carried out separately in the region annually. The broader study focused on 24 villages that were randomly allocated to one of three intervention arms: i) *Arm A* received both MDA and MDRV; ii) *Arm B* received MDA only; iii) *Arm C* received MDRV only. A deworming team (two community health workers and a nurse from the District Medical Office) delivered the MDA while a rabies team (a veterinarian and two field staff) delivered the MDRV. A comprehensive discussion of the methods used for the broader study and in-depth quantitative (and economic) analysis can be found in Lankester et al., 2019 [16]. This paper explores the social science elements of the study in more depth. The qualitative outcomes (including awareness of STH and rabies and attitudes towards our integrated interventions) are examined in further detail, including where they may align or contrast with the findings in the Lankester et al., 2019 paper.

Each intervention ‘event’ was delivered at the sub-village level (a smaller administrative unit within a village) using a ‘central-point’ delivery strategy. Following Tanzanian national regulations, children between 12 and 59 months were given an oral dose of mebendazole (250mg) and vitamin A (100,000 IU); children (and adults) over 59 months were given an oral dose of albendazole (400mg). Dogs brought into the MDRV clinics were vaccinated with a 1 ml dose of rabies vaccine (Nobivac, MSD Animal Health, Boxmeer, Netherlands).

### Evaluation of community perceptions about health interventions

To determine perceptions and attitudes towards health interventions, a post intervention socio-economic household questionnaire (survey), as well as in-depth interviews (IDIs) with village leaders and focus group discussions (FDGs) with a range of participants were carried out in 16 of the 24 villages. A team of two social scientists, under the supervision of the lead author, collected data between February–October 2016 (with data collection halted for two months during the rainy season). All interviews were audio recorded (when consented to by participants), and later transcribed and translated from Swahili or Maa into English by data collectors. Where interviews were not audio recorded, in-depth handwritten notes were taken by a dedicated note taker and typed for translation and analysis. Sixteen villages were chosen, as this was deemed an adequate number to provide depth of understanding into sentiments, opinions and perceptions of integrated delivery, and which did not need statistical significance. The survey explored knowledge about STH and rabies, household socio-economics and decision-making norms. The survey was based on, but not limited to, an extended knowledge, attitudes, and practice (KAP) style study [46] and included 10 randomly selected households per sub-village (3 sub-villages/village targeted) with 480 total surveys conducted. KAP based questions about STH and rabies were expanded to include questions about broader social issues, (e.g. norms around household dog ownership, participation in and perceptions of health interventions more broadly, and discussions about the role of leadership) within each site. Bringing more than 20 years experience working in this region with pastoralist communities, we were able to craft ethnographically informed data collection tools, and conduct informed observations and interactions with research participants. These added additional social and cultural insights, and provided depth in study design and data analysis, beyond the typical KAP study [25].

The IDIs (*n* = 59, 3.7/village) targeted community leaders (e.g. teachers, village government officials, health workers, elders) and consisted of a mix of open- and close-ended questions concerning public health interventions, personal behaviours around STH and rabies, and opinions of integrated intervention delivery. Additional questions explored the role of leadership and potential improvements for future health interventions. The FGDs (*n* = 30, approx. 2/village) targeted separate groups of men and women (due to cultural norms) and encouraged conversations about community health concerns and opinions about the integrated intervention strategy.

Social science analysis incorporates both the qualitative and quantitative data. We took two approaches to thematically code and analyse data: the FGDs and IDIs were coded and analysed using Nvivo ethnographic software, whilst IDIs were also coded and analysed using SPSS statistical software to summarize general thematic patterns. A general thematic coding framework emerged after reading through all 30 of the FGDs, which was then used to code in more depth a further 13 FGDs. The selection of 13 FGDs (6 women and 7 men) was purposeful, with the aim of selecting interviews that represented the breadth of the population of the research sites, reflected gender parity and represented the comprehensive scope of ideas across all intervention Arms. The broad coding themes included: awareness (and gaps) about opinions of, and attitudes toward integrated human-animal health interventions, constraints to participation, programme improvements and incentives, and drivers of health seeking behaviours, among others.

All 59 of the IDIs were analysed in-depth, both through their close-ended responses and by coding the open-ended responses and exploring the themes that emerged. IDI coding was done in two phases. After reading a selection of the IDIs, general thematic codes were developed and a coding dictionary was created which allowed a distillation of the themes. The IDI answers were then coded on a spreadsheet. Because the answers were based on open-ended, non-limited responses, using SPSS allowed the creation of multiple-response sets, similar to a multiple response-based survey. Finally, key ethnographic and qualitative responses were highlighted in both the FGDs and IDIs, which exemplify the emergent themes on key research questions.

### Evaluation of whether prior exposure to study methods facilitated a more favourable response regarding participation in integrated programmes

The outcome of the qualitative analysis prompted further exploration of the survey results to investigate whether prior exposure to integrated or non-integrated delivery impacted survey response. We carried out a post-hoc multinomial logistic regression analysis to compare survey responses from each Arm. This allowed us to investigate whether the intervention to which a respondent had been exposed (Arm A, B or C) impacted the likelihood of positive responses. The specific questions that were interrogated were i) would you be more or less likely to have your dog vaccinated if the vaccination was delivered through a programme that was integrated with a programme treating children for worms and ii) would you be more or less likely to bring your child for deworming if the treatment was delivered through a programme that was integrated with mass vaccination of dogs. This analysis bolsters the social science findings by indicating whether favourable opinions towards integrated delivery are impacted by prior exposure.

### Limitations of the research

It is well recognised that limitations and potential biases can arise in mixed methods qualitative health research. For example, ‘courtesy bias’, wherein respondents report what they think a health researcher wants them to hear, can occur, particularly in surveys [47–48] or ‘recall bias’ whereby participants may fail to remember past events [49–50]. However, as occurred in this study, these were countered by data triangulation (e.g. comparison of in-depth interviews, focus groups, and observations) as well as through careful translation, back translations, and knowledge of the cultural contexts for which data collection takes place (see Brown & Inhorn’s 2013 edited volume for discussions about strengths and limitations of qualitative health research [50]). Several additional aspects of our project provided confidence in our interpretation of data including our specific detailed ethnographic experience in the region, the general levels of trust built through authors’ affiliations with the local rabies vaccination programme, and the field team’s connectivity with the communities that resulted from social and familial networks. Such localised experience enhanced levels of trust and access for the enumerators and respondents alike.

### Ethics statement

All data were collected using an informed consent process. Participants volunteered to take part and gave either verbal and/or written consent. Verbal consent was sought for non-literate participants. Intervention participants under the age of 18 were consented through parents/guardians. The District Medical and Veterinary Offices in Ngorongoro District helped coordinate intervention implementation and informed community leaders about the project. Information sheets were provided to participants to take home (and for non-literate participants, they were encouraged to have a family member read it to them). Ethical applications for the study were approved by the National Institute of Medical Research (NIMR) in Tanzania (No. MR/53/100/353), and the Animal Care and Use Committee of Washington State University (04577–001). Further details about ethics and the consent process for the interventions can be found in Lankester et al., 2019 [16]. All names (individual and locations) were removed to protect privacy and maintain anonymity.

## Results

The findings presented in this paper centre on the qualitative outcomes and meanings around our integrated interventions. Economic and statistical evaluation of the data regarding feasibility and coverage of the intervention can be found in Lankester et al., 2019 [16]. This study expands on Lankester et al., 2019 in that its focus is on evaluating and highlighting where survey outcomes and qualitative data align and/or contrast. The key outcomes here focus on several key areas of exploration, including: awareness of Maasai community members regarding health issues related to STH and rabies; opinions and perceptions of health intervention programmes related to these diseases (particularly integrated human-animal intervention strategies, indicated in Box 1); and perceived constraints to, and means of improving, health intervention effectiveness (and by proxy, structural factors within health systems). Also, in the context of improving interventions, local opinions are highlighted about the role community leaders can play in the implementation of public health interventions.

**Box 1**

*It’s a good idea because we are getting two services at once and the reasons of these services is to prevent us from getting infections*.*—*Village leader, IDI 12

*To me I think it’s a good idea to combine [the programmes]*, *the problem will be if those doctors who are vaccinating dogs*, *if they are the ones who will provide worm drugs for human beings because how can you vaccinate dogs then at the same time you use the same hand to give medicine to people*?*–*Men’s FGD participant, Village 10

### Awareness of STH

“*Minyoo”* is the most common Swahili term for STH and is directly translated as ‘worms’. Thus, we have used the term ‘worms’ in the discussion of STH in the results. When village leadership were asked about the impact of worms on health, the IDI responses indicated high levels of awareness that worms cause health problems in people, despite only 27% of these respondents having experienced health problems associated with worms themselves. Although there were high levels of awareness among leaders, the responses across focus groups and surveys suggest that community members do not view worms as a cause of imminent or daily concern. For example, in the survey most respondents did not regard worms as a significant cause of health problems for people (see Table 1), and in FGDs (n = 30), when participants were asked to name their top five overall health concerns, worms were not given in any of the interviews.

**Table 1:** Survey and IDI questions regarding the impact of STH and which age groups are affected. S1 and S2 Data.

Interview type	Question	Results
Survey (n = 480)	Are worms a significant health problem?	38% YES
		62% NO
IDI (n = 59)	Are you aware of problems associated with worms in people?	83% YES
		17% NO
IDI (n = 59)	Can you describe what these problems are?	86% offered an open-ended response (detailed in text)
Survey (n = 480)	What age group/who do worms affect?	Children only: 46%
		Adults only: 1%
		Both children and adults: 27%
		Don’t know: 27%
IDI (n = 59)	Can you tell us who worms most affect (adults or children)?	Children only: 42% Adults only: 0% Both children and adults: 3% Don’t know: 54%

The IDI and survey results supported one another regarding general awareness of the source and the clinical signs of worm infection. Survey results presented in Table 2 revealed what participants believed to be key sources of worms (including food and soil) and avoidance methods (including properly cooking food).

**Table 2:** Household survey responses regarding sources and avoidance methods of STH infections. Participants were asked whether food and/or soil were a source of STH and whether cooking food, hand washing and wearing shoes were methods of avoidance. S1 Data.

Source & Avoidance Methods	Percentage of respondents (n = 480)
Food	82%
Soil	67%
Avoidance–cooking food	91%
Avoidance–hand washing	75%
Avoidance–wearing shoes	36%

Regarding health impacts of worms, 85% of survey respondents understood that worms cause “stomach illness” when asked specifically about symptoms. An open-ended question was also asked in the IDI about “problems associated with worms” (Table 1). The open-ended answers contained a wide array of information (Table 3) with 22 unique responses given by all but eight of the respondents. While four of these unique responses related to *sources* of worms (including consumption of contaminated food or water, touching contaminated things, children eating soil, and non-hand washing before eating), the remaining unique responses were about the health problems worms caused. All respondents who offered an answer to this open-ended question (86%) had at least one health issue named in their response including digestive or gastrointestinal issues (see Table 3 for details).

**Table 3:** Health problems of STH from IDI. Table 3 represents the variety of open-ended responses related to the questions, “are you aware of problems associated with worms in people?” and “can you describe what these problems are”. While 51 of 59 respondents offered an answer to this question (e.g. 86% overall), responses were open-ended and multiple answers were given. The percentages offered in the second column represent the percentage of respondents (of 51) who offered this answer. S2 Data.

Health problems associated with worms	Percentage of respondents (n = 51)
Stomach pain	39%
Loss of appetite	36%
Diarrhea	19%
Bloated belly	17%
Nausea	15%

### Awareness of rabies

Awareness of rabies was primarily assessed through the survey and IDIs (Table 4). Respondents (76% survey and 100% IDI) were aware of rabies as a health issue and nearly all (98%) survey respondents understood that dogs were the species that was most commonly affected. Of concern, however, was that, despite on-going rabies campaigns, more than half (62% survey and 58% IDI) had direct experience with rabies within their community with 80% of survey respondents and 56% of IDI respondents being dog owners, or having one in their compound. Awareness that animal species other than dogs are susceptible to rabies was relatively low with only 26% aware that rabies can be transmitted via wildlife. Respondents reported being concerned about rabies, with 54% and 55% reporting that they were very concerned about rabies in dogs and in people, respectively.

**Table 4:** Awareness of Rabies. The outcome of ‘awareness’ based questions about rabies in the survey and IDI. Questions asked are listed in the first column. S1 and S2 Data.

Question	Interview Type–Survey (n = 480); IDI (n = 59)	Results
a. Do you own dog(s)	Survey	YES 80%
	IDI	YES 56%
b. Have you heard of rabies?	Survey	YES 76%
	IDI	YES 100%
c. What are the key species affected?	Survey	DOGS 98%
	IDI	NA
d. Can rabies be transmitted via wildlife?	Survey	YES 26%
	IDI	NA
e. Do you have direct experience with rabies?	Survey	YES 62%
	IDI	YES 58%
f. How can rabies be transmitted? (to dogs)	Survey	DOG BITES 94%
	IDI	NA
g. How concerned are you about rabies in dogs in your community?	Survey	Very- 54% Moderate– 40% Not at all concerned– 7%
	IDI	NA
How concerned are you about rabies in humans in your community?	Survey	Very– 55% Moderate– 35% Not at all concerned– 10%
	IDI	NA
h. Have you ever vaccinated your own dogs for rabies?	Survey	YES 38%
	IDI	YES 79%

### Does awareness equal participation?

Despite 94% of survey participants being aware that being bitten by a rabid dog is a method of transmission, only half (50%) believed that rabies in people can lead to death, whereas 38% did not know if it led to death. We have no follow up data about why they believe this. While 62% had direct experience of rabies within their communities, only 38% of survey respondents reported ever having vaccinated their dogs, despite MDRV being offered for free in these communities. This is less than the proportion of village leaders who, when interviewed, reported high levels of awareness of rabies (100% of IDI), of vaccination programmes (78% of IDI) and having vaccinated their dogs (79% of IDI).

Explanations given for why survey respondents did not participate in MDRV hosted in their communities included: dogs refused to go (35%); owners were otherwise occupied herding cattle during the event (27%), and owners were not aware of the MDRV programme at the time (8%). Despite relatively few survey respondents acknowledging their dogs had previously been vaccinated, survey results estimated that 67% of dogs in the target villages were vaccinated through the MDRV campaign offered by this project.

In all interview platforms, the majority of people were familiar with the need to seek treatment following dog bites to protect themselves against rabies, with 77% of survey respondents correctly specifying human vaccination (with post-exposure prophylaxis) as the treatment needed. The need to seek medical treatment following a bite from a dog was clearly summarised by women in a focus group presented in Box 2:

**Box 2**

*Respondent 1*: *Yes we have seen [rabies] since last year*. *But normally after getting this disease*, *that dog is going to be killed in order to avoid any impact that may affect people or livestock*. *Interviewer*: *Do you know people in your community who have had rabies*? *Respondent 2*: *No*, *there is not anyone who has gotten rabies in our area*, *although dogs they are biting people*, *but those people they have been taken to the hospital in order to prevent the person from dying because of rabies*.*–Women’s FGD participant*, Village 2

The high costs of PEP were a key concern around rabies (as the quote in Box 3 below demonstrates), yet there was recognition that dog vaccination offers the best option in reducing disease risk.

**Box 3**

*Respondent 1*: *We have seen people bitten by the dogs but we were not sure if that dog had rabies or not but for those cases many of them normally are rushed to the hospital for the treatment*. *Respondent 2*: *I don’t understand why our government lets this rabies treatment [human PEP] be very high cost and it’s difficult for most of the people to accommodate it*, *especially for us poor people*. *Interviewer*: *Yes it’s true the costs may be high but it’s important now when you see these rabies campaigns it is better we take that opportunity by bringing our dogs for vaccination*. *Together we can make it happen*, *by destroying this disease for good*. *Respondent 1*: *A dog should be vaccinated after how long*? *Interviewer*: *Dogs should be vaccinated every year*.*—*Men’s FGD participant, Village 3

### Opinions of health intervention campaigns

In response to questions about public health programmes, the majority of respondents in the IDI were aware of health programmes (outside of our intervention) that had occurred in their communities within the last year (51%) or within the last five years (59%). Further, 58% of IDI respondents had participated in previous programmes and all viewed them favourably. The qualitative data collection on health campaigns allowed a deeper exploration of viewpoints and opinions about public health programmes and demonstrated how issues around health benefits, healthcare access, and cost are interconnected. For example, in 92% of FGDs (n = 30), people expressed positive opinions about public health campaigns because they provide positive health benefits. This is exemplified through statements such as that they “reduce health problems”, “prevent infections” or “provide access to needed medicines or vaccines” that were “otherwise unavailable”. When asked ‘do people think that public health/animal health campaigns can help them with their personal or household issues?’, there was general agreement across respondents, with reduction of household health costs and provision of information or education about health issues frequently mentioned as benefits (see Box 4).

**Box 4**

*Yes*, *it’s helping us a lot especially because the place we are living is far from the hospital and we were given free treatment as well*.*—*Women’s FGD participant, Village 6

*Yes*, *it’s really helping us greatly by preventing these diseases*.*—*Men’s FGD participant, Village 8

*Yes it has many benefits because we Maasai*, *we are not using this medicine*, *but we hope that this drug has lots of benefits*.*—*Men’s FGD participant, Village 10

### Opinions on integrated health delivery programmes: Benefits and constraints

Because the delivery method varied across study Arms (A-C), we breakdown the responses regarding opinions of integrated health delivery accordingly. When asked for opinions about participating in integrated health delivery programmes, the majority of survey respondents in all Arms stated that they were more likely to get their dogs vaccinated if child deworming was delivered with it, or vice versa (Table 5).

**Table 5:** Likelihood of participating in an integrated health delivery programme. The survey questions and the Arm specific responses are given. Arm A received both dog vaccination and deworming for children. Arm B received deworming for children only and Arm C received dog vaccination only. S1 Data.

Survey Qs—*More* or *less* likely to participate in joint intervention:	ARM	Response	Frequency	% (n = 480)
To get **dog rabies vaccination** if delivered **with** child deworming	A	More	202	79%
To get **dog rabies vaccination** if delivered **with** child deworming	B & C	More	135	63%
To get **child dewormed** if delivered **with** dog rabies vaccination	A	More	186	73%
To get **child dewormed** if delivered **with** dog rabies vaccination	B & C	More	125	58%

When explaining *why* they would more likely participate in integrated interventions, qualitative interviewees explained their reasoning (see Box 5):

**Box 5**

*That’s a good idea [integrated programmes] because we are getting two services at once and the reasons of these services is to prevent us from [getting] infections*. *As we heard that rabid dogs are very dangerous to humans and once you are bitten*, *with no treatment [available] you will die after a few days*. *So combining these two events is not a problem because the important thing is we are getting good health services for our wellbeing*.*—*Men’s FGD participant, Village 1

Within the FGDs, all expressed positive responses about integrated interventions with reasons ranging from efficiency, effectiveness, cost saving, to health benefits. Nearly all the FGDs (92%) thought the integrated programmes would reduce health problems in their families and their animals, 85% thought they were saving time or effort (getting “two for one” health treatments, “saving time”, “saving effort”, and/or “reducing costs”), whilst 73% believed they were receiving worthwhile vaccines and drugs and residual health benefits as part of the integrated platform. When asked about the combination of STH and rabies responses were often similar to those given in Box 6:

**Box 6**

*Respondent 1*: *Yes*, *it’s helping us a lot by people becoming aware of these diseases*, *also it prevents us (from getting them) by decreasing these rabid dogs cases*, *and people being (more) aware of the importance of these health programmes*. *Respondent 2*: *Our children are prevented from getting worm infections by getting free treatment*.*—*Men’s FGD participant, Village 3

*Interviewer*: *What kind of challenges may occur if they are integrated*? *Respondent*: *I don’t think if there are any challenges by combining them because we get two services at the same time*. *You bring your dog for vaccination and at one point you take the medicine (deworming)*. *To be honest combining those somehow reduces*, *(its less) time consuming*.*—*Men’s FGD participant, Village 8

*Respondent*: *That’s the best idea and we are willing to participate because we are getting two health services at the same time*. *The important thing here you should differentiate those doctors depending on the programmes*, *like if he/she distributes worming drugs it’s only that and (they are) not mixing things*.*—*Women’s FGD participant, Village 6

When asked about whether respondents would likely participate in future integrated programmes, 84% of IDI respondents stated they were “more likely” to bring a child to get deworming medication if delivery was integrated with other health programmes, and 86% were “more likely” to bring their dogs to get vaccinated if interventions were integrated.

Does prior exposure facilitate a more favourable response regarding participation in integrated programmes? The data suggests it does, with participants who participated in the integrated programme (Arm A,) expressing less doubt in FDGs and IDIs than those in the non-integrated programme (Arms B and C,) as demonstrated below in Boxes 7 and 8 respectively.

**Box 7**

*Interviewer*: *What do you think are the strength and weakness of combining rabies and mass worming programme*? *Respondent*: *To me there is no weakness but there is a lot of strength because from the time when this vaccination started we didn’t see again rabid dogs and also for the human being it will help to reduce these problems of worms that have been another problem for people*.*—*Men’s FGD participant, Village 18, Arm A

*Respondent*: *It has a lot of benefit because since the start of rabies control programme it’s has been very rare to get the dog who has rabies disease and also worms drugs it will help combat worms within human body for both children and adult but no any weakness at all*.*—*Women’s FGD participant, Village 2, Arm A

**Box 8**

*Interviewer*: *Do you think the response for getting people to participate in these programmes would be more effective together*? *Respondent*: *Response will be low because of combining them*. *It will be good if they can be scheduled in different days like one can be started after another*. *Combining them is not hygienic like touching dogs then at the same time you get a treatment*. *[I] will be vaccinated but taking the treatment at the same point will be difficult because I need to wash my hands*.*—*Retired village chairperson, IDI 17, ARM C

*Interviewer*: *What do you think about the idea of combining programmes for rabies vaccination of dogs with mass worming treatment of humans*? *Respondent*: *I believe it will be a good exercise although I have never seen it before and its new to us*, *so I can’t say anything more on that*.*—*Respected elder, IDI 9, ARM B

To investigate this further, we carried out regression analysis to determine whether the likelihood of the positive responses given in Table 5 were impacted by the Arm of the study that the respondent was in. The results of this analysis (Table 6) indicated that the odds of a respondent from the integrated Arm A stating that they would be “more likely” to bring their dog for vaccination if the programme was integrated with child deworming were increased, when compared to responses from Arm B and C. This increase in odds was significant at the 5% threshold when Arm A was compared to Arm B (p = 0.009). Similarly, the odds of a respondent from Arm A stating that they would be “more likely” to bring their child for treatment if the programme was integrated with dog vaccination were increased, when compared to responses from Arms B and C, and significantly so when compared to Arm C (p = 0.03). Furthermore, the probability of respondents from Arm B giving the response that they were “less likely” to bring their dogs or children if the programme was integrated was higher when compared to Arm A, significantly so for dogs (p = 0.016). And the probability of respondents from Arm C giving the response that they were “less likely” to bring their dogs or children if the programme was integrated was also higher when compared to Arm A, significantly so for both dogs (p = 0.02) and children (p = 0.036). These regression results provide quantitative support to the social science findings that respondents who participated in the integrated Arm A express more favourable opinions towards integrated delivery than those who took part in the non-integrated Arms (B and C).

**Table 6:** Likelihood of participation in joint programme. The output of multinomial logistic regression analysis investigating whether the experience of the integrated programme impacted survey responses. The two survey questions that were analysed asked i) whether respondents would be more or less likely to have their dogs vaccinated if the delivery was integrated with the child deworming (Question = Dog) and ii) whether respondents would be more or less likely to bring their children for deworming treatment if the delivery was integrated with the rabies vaccination (Question = Children). Arm A was the baseline factor against which Arm B and C were compared. The odds ratios (95% confidence intervals and p-values) for the likelihood of the responses in Arm B and C, as compared to Arm A, are given. Arm A received both dog vaccination and deworming for children. Arm B received deworming for children only and arm C received dog vaccination only. S1 Data.

Question	Response	(Intercept)	Arm B Odds Ratio (95% CI) p value	Arm C Odds Ratio (95% CI) p value
Dog	Less likely	0.059	5.31 (1.35–20.78) p = 0.016	5.04 (1.23–20.56) p = 0.02
Dog	More likely	3.96	0.50 (0.29–0.84) p = 0.009	0.66 (0.39–1.14) p = 0.13
Child	Less likely	0.03	10.65 (2.2–51.53) p = 0.003	5.63 (1.12–28.44) p = 0.036
Child	More likely	2.82	0.79 (0.48–1.31) p = 0.36	0.48 (0.29–0.78) p = 0.03

### How genuine are favourable opinions about integrated delivery?

While there was considerable support for integrated delivery across the Arms of the study, these affirmations were embedded within discussions around both constraints to, and incentives for, participating. A thorough analysis of the qualitative data allowed more subtle opinions about the delivery methods to be recorded. It appears there is less clear-cut optimism regarding integrated human–animal health delivery than initial analysis of the interviews would suggest, with all participants discussing various “challenges” of integrated delivery. For example, where a positive comment was provided, it was often followed by suggestions to “improve” delivery. A key concern related to the central-point method of vaccine delivery for dogs (see Boxes 9 and 10), with 14% of IDIs viewing bringing dogs to a central point in the village as a constraint. Similarly, the majority of FGD respondents (62%) expressed preference for house-to-house (‘*boma*’ or compound of households) delivery methods for rabies vaccination (i.e. not the deworming component of the campaign), citing concerns about dog/herder availability (54% FGD) and how easy it would be to coax dogs to the central point site (9% FGD).

**Box 9**

*I agree for rabies and worming programme to be integrated*. *But for dogs*, *you better go one boma after another because it’s very challenging to bring dogs in the central point because they won’t come and it’s difficult to catch them but for human being no problem*.*—*Men’s FGD participant, Village 18

**Box 10**

*Respondent 2*: *…you can take both to go to be treatment (children and dogs) but the challenge comes when the dog refuses to follow you and sometimes it’s difficult to catch so that they can be vaccinated*.*—*Women’s FGD participant, Village 16

Other comments reflected concerns about the hygiene implications of combining human and animal health delivery, with concerns about staff using “the same hand” to deliver medicines to people after having dealt with dogs (thus there was some hesitancy to receive treatments together (38% FGD). Others expressed the need for wanting “more information”, “more education”, or time to think more about the integrated programme options (10% IDI; 7% FGD).

### Barriers to participation

Finally, there were a number of potential contextual barriers that respondents stated that they faced in participating in public health programmes. The primary barriers mentioned in the survey included distance (74%) and time (61%), followed by cost, lack of transportation and the requirement for spousal approval (see Table 7).

**Table 7:** Survey responses to barriers to participating in public health events. S1 Data.

Main barriers to you participating in public health events	In a Yes/No response, the % answering ‘yes’ (n = 480)
Distance	74%
Time	61%
Lack of public health events in their community	23%
If spouse allows	15%
Money	17%
Transport	13%
Trust	9%
Whether family participates	3%
Whether neighbours participate	3%

The discussions about barriers to participating in health events in IDI and FGDs were less direct than the questions posed in the survey, and the mention of these barriers tended to occur more loosely during conversation about this project’s specific intervention. These included issues around communication (not enough community awareness) and geography (location, environment, distance, transportation) as well as issues of trust (in drugs and in those providing the intervention/campaign). Both IDI and FGD respondents suggested that a key barrier to participating in public health programmes more generally was the general absence of public health programmes or health care in their communities in the first place. In response (Box 11) to a question about public health programmes in their community, one participant responded:

**Box 11**

…*even if they come they won’t provide to everyone in the whole village*. *You can find if they come it’s just a few people who get the drug… Even today a lot of mamas are happy because we don’t (normally) get any services at all in our area*.*—*Sub village leader, IDI 59

### Means of improving health interventions

Respondents offered suggestions regarding how participation in the campaigns in their communities might be improved. FGD participants suggested that provision of information about the event well in advance of it being implemented (69%) and provision of education to improve community awareness about the diseases and the effects of the drugs (54%) could all help to gain support and improve participation (Box 12).

**Box 12**

*A good incentive to make all people participate is that first of all we are requesting to be given information before these people who provide the drugs come.—Women’s FGD participant, Village 1*

*I was thinking a good motivation is to educate them first about the programme, like about the importance for them to participate and how they will benefit from that participation and what they will lose if they won’t participate in the programme. Awareness is something very important in our village to make them believe and willingly to participate, otherwise it will be very difficult for them. -Women’s FGD participant, Village 2*

*To me I think it’s better before you start the programme that you go to the villages and provide education about what is the importance of this programme and for how long they are supposed to take these worm drugs so that they may know that even after this programme they may go to buy this medicine themselves when they see signs/symptoms of these disease called worms*.*—*Village Leader, IDI 9

The important role that community leaders can play, providing information and education for health interventions and mobilising the community, was discussed in the majority of FGDs (62%) (Box 13).

**Box 13**

*Respondent 2*: *because they* [leaders] *are ones who*, *when they provide information to people*, *they are respected*. *They are also listened to by people due to the positions they have*, *and they have already shown good commitment*. *Respondent 1*: *It’s because they have been selected to do those responsibilities so as to make sure that people they get all the required services*. *Respondent 2*: *Also to me it’s both sub village leaders and chairman of the village because they are the ones who can easily provide information to people*.*—*Women’s FGD participant, Village 1

Finally, there was strong concurrence across all of the IDIs that the leaders themselves would be the most effective people within the community to communicate with, and garner participation from the members of their own communities.

## Discussion

Our results suggest that integrated One Health approaches are a viable option for delivering health interventions to hard-to-reach communities, and that qualitative social science can highlight why this is so. We demonstrate that careful consideration of local experiences with such programmes are an important component to acceptance and successful delivery. The use of a qualitative approach provided depth of understanding behind seemingly positive responses to integrated interventions, revealing concerns around hygiene, modes of delivery, and the need for more information. Structural health problems also came to light, where participants welcomed combined interventions due to lack of access to health services more generally. Thus, foregrounding social science in NTD interventions can help uncover complexities and nuance in local perceptions, awareness, participation and attitudes towards integrated delivery which may lead to more effective outcomes.

### Awareness and participation

Levels of awareness of both STH and rabies were high across all communities in the study, however there were disparities between the awareness of community leaders and non-leaders, with more awareness and concern about NTDs coming from leaders. These disparities are unsurprising, and likely stem from community leader’s participation in previous health campaigns, organising events with higher level government officials or NGOs, and potentially because they are often the first line of reporting for rabies or other health related incidents. This may also explain why leaders were more likely than non-leaders to state that they regularly vaccinate their dogs against rabies. Additionally, while nearly half of all participants erroneously believed that STH affected children only, this seems likely due to the school-children centered national campaigns that emphasize children as affected by STH and as targets for these interventions.

While analysis of gender differences in response did not yield any notable differences in awareness, we note that one of the key barriers to participation included *spousal approval*. Given the relegated role of women as household decision makers in Maasai communities, this issue likely stems from women needing their husband’s or father’s approval before participating in such events. Given women are the primary caretakers for children, often overseeing their health care and managing health seeking for their children in these communities [51], future interventions should pay careful attention to these decision-making norms and potential gender disparities in accessing public health goods.

Community leadership was utilised in every village targeted by the programme to communicate the details of the interventions with the community members and, in support of this, conversations with leaders and non-leaders alike point to the importance of community leaders as intermediaries for interventions. This, in conjunction with higher levels of awareness and participation, indicates that leaders have an important role to play facilitating such interventions. However, the disparities identified by this study between awareness and participation among leadership and community members suggests further work needs to be done to support leaders to ensure key messages reach the wider community.

### Benefits of integrated delivery

Despite the epidemiological pathways associated with STH and rabies being separate, there appears to be value in integrating interventions through a One Health approach not only because integration leads to cost savings that can be realised while meeting effective rates of coverage [16] but also because of the beneficial outcomes to people and their animals and the positive attitudes people have about integration. As noted, the costs for delivery of integrated platforms is lower than individual projects, however ‘cost’ saving should not be the only factor for promoting integration. Time saved (up to 33% less time to participate in an integrated programme [16] was an additional benefit, and one that contributed to strong positive opinions about the project. While this can be viewed as an ‘economic’ benefit, the enthusiasm and support also reflected appreciation for service providers for considering participant’s busy lives as the qualitative data demonstrates. Likewise, that attention was paid to communities that are often neglected in public health outreach was recognised by communities who saw that health matters of concern were being considered.

Furthermore, in addition to cost savings, respondents often equated benefits of interventions more broadly to access to essential medicine and receiving health information. These emerged as key themes as evidenced by people’s comments about their experiences and views of the integrated intervention. While some structural barriers to health in this region may not be solved solely by integrated programmes, our programme demonstrated that when people are provided access to health care (both veterinary and human) *and* information, gains in coverage, and future willingness to participate in public health interventions may occur. If offering a cost-effective integrated programme can be beneficial on many levels, it can only do so by addressing the stated concerns about integration which could hamper uptake. Providing a means for addressing and overcoming those challenges will come from integrating the ideas and efforts of all stakeholders, from researchers, leaders and community members alike. Interestingly, given the coverages achieved across the three Arms of the study were similar [16] the stated challenges did not appear to dissuade people from participating.

### Attitudinal and behavioural complexity

Survey data revealed overall positive attitudes towards integrated interventions, which when supported by qualitative data, provides deeper insights into how people perceive integrated interventions. These include insights into w*hy* people appreciated the time and cost savings, *what* kinds of benefits they received overall, and *how* they could overcome challenges. However additional care needs to be taken generally when interpreting survey data in both OH and general public health studies. For example, does the output of the regression analysis suggest that integrated delivery was perceived to have worked well? That is, were people who experienced it more enthusiastic about integrated health delivery? Or does the regression output reflect a research bias caused by participants aligning answers to what they think the enumerator wanted to hear? In combining the insights from the qualitative data, where respondents clearly stated preference for effective programmes that save time and money, and which led to perceived positive health outcomes (and attention paid to communities often neglected in public health outreach) we believe the regression analysis does reflect a positive impact of experience of integrated delivery, and one that led people to be more likely to support these interventions in the future.

A closer exploration of qualitative data did also reveal, however, how overall positive sentiments are not as clear cut and ‘positive’ per se. Quantitative responses (close-ended, yes/no or direct preference questions) often fail to reveal why programmes may succeed or fail even if it appears most participants agreed they would participate. Through exploring qualitative data it becomes apparent where difficulties in attending integrated interventions lie. For example, IDIs and FGDs revealed concerns relating to hygiene, mode of delivery (i.e. bringing dogs to a central point), hesitancy to receive treatments together, needing more information/education about the intervention and needing more time to consider. Yet these did not necessarily hamper participation outright, demonstrating the complicated choices people make and where perceived benefit seems to outweigh concern. Thus, qualitative data gathered throughout this study provided a deeper insight into how people perceive integrated programmes. Through explaining what kinds of awareness and understanding people have of these NTDs of concern, future targeted interventions can benefit from learning where to focus information exchange or where to provide exposure of health solutions to individuals and communities.

## Conclusion

As Hinchliffe (2015: p. 34) [52] states, “objects of disease are never straightforward” and neither are the potential responses and outcomes to health interventions that tackle them. In this pastoralist region of northern Tanzania, much like throughout pastoralist and agro-pastoralist areas of East Africa, human and veterinary health services are often limited. Key findings from this study are likely to have broad applicability to a range of low-income settings where provision of human and animal health services is limited, including many areas of sub-Saharan Africa. For example, many people in under-served communities are likely to recognise and appreciate efficiencies and efforts to improve health delivery. The finding of potential concerns around hygiene in relation to shared human-animal delivery is also likely to be as important (or even more so) in other communities which have less contact and familiarity with animals and animal health interventions. Thus, we demonstrate how an integrated, One Health approach to complex health problems can highlight critical issues that influence the success or failure of NTD interventions, including a consideration of systemic health issues and local perceptions of mode of delivery. Qualitative research, in particular, reveals that perceptions of integrated interventions are nuanced and complex and may offer insight into how to build more effective interventions in the future.

## Supporting information

S1 Data Survey Data. (XLSX)

S2 Data IDI Data. (XLSX)

S3 Data FGD Data. (DOCX)

S4 Data FGD Question Guide. (DOCX)
