# Supplementary material for: “Using the same hand”: The complex local perceptions of integrated one health based interventions in East Africa
Source: PLoS Negl Trop Dis. 2022 Apr 4;16(4):e0010298. doi: 10.1371/journal.pntd.0010298 (PMC9009769; doi:10.1371/journal.pntd.0010298)
Supplement: S3 Data — (DOCX) [file pntd.0010298.s003.docx]

**Supplementary FGD data for evaluating combined STH & rabies interventions in Northern Tanzania. Data should not be distributed without author's approval.**

**Thematic codes and data summary for focus group discussion data**

**Key questions analysed (see supplementary FGD question guide):**

- Do you think (worming) program was effective at reaching adults?
- What are the challenges in getting children to participate (worming program/rabies program)?
- *How do you think people will respond to combining a rabies vaccination event with a worming event?*
- *Do you think people would be likely to participate if these events were combined or not?*
- *Do you think there are strengths and weaknesses of combining rabies and mass worming programs? What are each?*
- *What do you like/dislike about the program/methods?*
- *What do you think by integrating these two programs in your community?*

| **Response ***denoted by key phrases and terms (as stems and derivatives) | **Key terms/phrases used to connote** |
| --- | --- |
| **Positive** response = favourable to idea, concept, practice, behaviour, event | - “good idea” - “I like” - “good” - “I prefer” - “benefit” - “provides” … (education, information, good health outcome) - “reduces problems” - “opportunity” |
| **Negative** responses = unfavourable to idea, concept, practice, behaviour, event | - “difficult” - “challenge” - “unhygienic” - “do not like”/”dislike” - “harmful” - “missed” (missed opportunities, participation) |
| **Neutral** responses = neither positive nor negative, often respondent in need of more information | - “more information needed” - “good and bad”/neither good nor bad - “middle” |

| Themes & Codes (broad coding themes are top tier and in bold, with sub themes listed underneath) |
| --- |
| **Social influences** |
| Education is needed about the program |
| Leadership (to take a larger role) |
|  |
| **Belief and consequences** |
| Mistrust |
| Trust |
| Side effects of the drugs |
|  |
| **Health Seeking Behavior** |
| Drug shops |
| CHWs |
| Vet shops |
| Livestock officers |
| Hospital |
| Health campaigns |
| Mixed treatment |
| CAHWs |
| Traditional healers & herbs |
| Government sponsored |
|  |
| **Constraints** |
| Travel distance, no transport |
| Cost |
| Distance to site |
| Barriers of access and availability |
| Lack of infrastructure |
| Human & animal treatment together |
| Dogs & kids unavailable |
|  |
| **Awareness or knowledge** |
| Awareness of intervention programs |
| Susceptibility and severity |
| Effects on children |
| Awareness of signs & symptoms |
| Causes & prevention of disease |
| Lack of knowledge |
|  |
| **Community response on combo program--positive** |
| Program effectiveness |
| Acceptability-accepted, good for the health of the family |
| Perceptions by appreciated the program, beneficial, effectively, capacity of reducing & destroy the diseases |
| Health benefit- vaccine and drugs |
| Two for one save time spent |
|  |
| **Sustainability** |
| Available at any time-continuously available yr after yr |
| Accessible |
|  |
| **Lack of awareness or knowledge** |
| Credibility of information sources |
| Difficult to explain about disease |
| Causes, symptoms & signs of diseases |
| Limited awareness demonstrated about diseases/health issue |
| Lack of adequate information available about programs |
|  |
| **Benefit of public health campaign** |
| Reduces treatment costs |
| Provides information |
| Economic incentives |
| Reduces health problems |
| Good communication |
|  |
| **Community response on combo program--negative** |
| Mistrust & fear due to previous interventions |
| Lack adherence with treatment regimes |
| Difficult method used |
| Boma to boma preferable |
|  |
| **Program improvement & incentives** |
| Improved drug supply within village |
| Affordability |
| Improved communication at all levels of campaign |
| Information sharing (2) |
| More publicity is needed to encourage participation |
| Early information about the program schedule |
| Boma-to-boma |
| Education |
| Community awareness of disease & drugs |
| Leadership communication |
|  |
| **No Answer** |

Positive response coding references in FGDs

| **Node (denotes a ‘code’ in Nvivo ethnographic software)** | **Number of interviews coded at this node (N=13)** |
| --- | --- |
| Health benefit- provides vaccines and drugs | 10 |
| Reduces health problems | 12 |
| Two for one– save time spent | 11 |
| Boma-to-boma preferable | 9 |
| Acceptability-accepted, good for the health of the family | 9 |
| Program effectiveness | 7 |
| Benefit of public health campaign-GENERAL | 5 |
| Community response on combo program – positive—GENERAL | 4 |
| Provides information | 3 |
| Good communication | 2 |
| Economic incentives | 2 |
| Reduces treatment costs | 2 |

Negative response to mixing interventions

| **Node** | **Number of interviews coded at this node (N=13)** |
| --- | --- |
| Difficult method used (i.e. central point difficult) | 11 |
| Boma to boma preferable | 9 |
| Belief and consequences\Mistrust | 5 |
| Negative-general | 4 |
| Lack adherence with treatment regimes | 0 |
| Mistrust & fear due to previous interventions | 0 |

Key Constraints to participating in intervention:

| **Nodes** | **Number of interviews coded at this node (N=13)** |
| --- | --- |
| Barriers of access and availability | 7 |
| Dogs & kids unavailable (herding) | 7 |
| Human & animal treatment together | 5 |
| Constraints-GENERAL | 2 |
| Distance to site | 2 |

Key incentives to participate in (public health) programs

| **Nodes** | **Number of interviews coded at this node (N=13)** |
| --- | --- |
| Providing early information about the program schedule | 9 |
| Shifting to a boma-to-boma model | 8 |
| Leadership communication (leaders communicating with community, acting as liaisons etc.) | 8 |
| Community awareness of disease & drugs (improving) | 7 |
| Providing additional Education | 4 |
| Program improvement & incentives-GENERAL | 3 |
| Improved drug supply within village | 3 |
| Improved communication at all levels of campaign | 2 |
| Affordability-making it affordable (or the drugs affordable) | 2 |
| Information sharing | 1 |
| More publicity is needed to encourage participation | 1 |
