# Supplementary material for: “Using the same hand”: The complex local perceptions of integrated one health based interventions in East Africa
Source: PLoS Negl Trop Dis. 2022 Apr 4;16(4):e0010298. doi: 10.1371/journal.pntd.0010298 (PMC9009769; doi:10.1371/journal.pntd.0010298)
Supplement: S4 Data — (DOCX) [file pntd.0010298.s004.docx]

1. Would you please describe 5 important health concerns in this community. (What are 5 main health issues that concern you in your community).
2. * *Have there been any public health/treatment programs in your community in the past year? Can you describe them to us? (If they do not answer the following in their description, please ask the following):*
   1. *What events occurred?*
   2. *Where in the village did they hold these events?*
   3. *Who sponsored them?*
   4. *Who were target participants? Did youparticipate? (why/why not)?*
3. Have you heard of something called “worms” [*minyoo*]? Can you tell us what you have heard about worms?
4. What kind of problems do they cause? Can you describe what kinds of health problems for people are caused by worms? *Can you describe the symptoms for the health problems worms can cause in people?*
5. *Can you explain where worms in people come from?*
6. What are the ways to prevent this?
7. Who is most susceptible to worms, adults or children?
8. Can adults get sick from worms also?

*This question is to be asked if they do not name a worming program in question 2. If they named the worming program, then please ask about the program they mentioned using the follow up questions a-d below.*

1. *Can you tell us about any worming programs that hascome to your village to treat children in the past year (few years)?*

*Ask the following as a follow up questions if they do not explain some of these issues in their description of the program*

1. *If so, who runs/sponsors these programs?*
2. *When were they last in your community?*
3. *Is this a government program?*
4. *What age children did they treat?*
5. We are going to ask you a few more questions about the worming program that came to your village:
6. *Do you think that this program (for treating worms) has been effective at reaching all/most children in your community? Why /why not?*
7. *Do you think this program was effective at reaching adults? Why/why not?*
8. *Do you think people like participating? Why/Why not*
9. *Do you like/not like participating? Why/Why not*
10. *What are challenges in getting children to participate?*
11. *What are challenges in getting adults to participate?*
12. *What would be a good incentive to get people to participate?*
13. *Please share any suggestions about improving the worming program, especially for reaching older children and adults*
14. *(KEEP this discussion brief) *Does a vaccination team come to your village to vaccinate dogs? Who are they? How often do they come? What do they do here?*
15. *Please tell us what you think of the rabies vaccination program that comes to your village to vaccinate dogs. (If they offer no opinion, ask the following):*
16. *Do you like the program/methods? Why/why not?*
17. *Do you think they are effective at communicating the reasons for vaccination?*
18. *Do you think they are able to reach everyone who needs vaccination in your community? Why/why not?*
19. *What challenges do you think they have?*
20. *Have you seen rabid dogs or other animals in your community in the past year?*
21. *Please explain*
22. *Do you know any people who have had rabies in your community?*
23. *What happened? (Add prompts: Was it an adult or a child affected? Did they get taken to the hospital? What happened to their health? Etc.)*
24. *How do you think people will respond to combining a rabies vaccination event with a worming event? Why do you think people would be likely to participate or not participate if these events were combined? [if not answered in above, follow with: Do you think that combining rabies vaccination of dogs with mass worming treatment of humans is a good idea?*? *Why or why not?]*

These may be answered above [if so skip]

1. *What do you think are the strengths and weaknesses of combining rabies and mass worming programs? (If no answers are provided, ask the following*)
2. *Do you think people would be more likely/less likely to participate if they are combined? Why/why not?*
3. *Do you think it would help communication/informing people if these programs were combined? If so, why?*
4. *Do you think there would be less/more repetition if they were combined? (Do people get tired of having public health events/development projects in your community?)*
5. *What kind of challenges may occur if they are combined?*

(This may be answered above, be mindful not to repeat)

1. *Are people/more or less likely to get their/children and themselves/ wormed if the program is delivered with a rabies control program?*
2. *Please explain why?*
3. *Do you think people in the community are more or less likely to get their dogs vaccinated if the program is delivered with a worming control program?*
4. *Please explain why/why not?*
5. *Are there local medicines that can treat worms/stomach ailments?*
6. *What medicines do people prefer? Local or doctor based?*
7. Why do people prefer these?
8. *Who can offer advice people will listen to about participating in public health/animal health campaigns? Why are they respected/listened to?*
9. *Do people think that public health/animal health campaigns can help them with their personal/household health issues? How can they help them?*
